# Circulating Tumor DNA as a Prognostic Determinant in Small Cell Lung Cancer Patients Receiving Atezolizumab

**DOI:** 10.3390/jcm9123861

**Published:** 2020-11-27

**Authors:** Guillaume Herbreteau, Alexandra Langlais, Laurent Greillier, Clarisse Audigier-Valette, Lionel Uwer, José Hureaux, Denis Moro-Sibilot, Florian Guisier, Delphine Carmier, Jeannick Madelaine, Josiane Otto, Pierre-Jean Souquet, Valérie Gounant, Patrick Merle, Olivier Molinier, Aldo Renault, Audrey Rabeau, Franck Morin, Marc G Denis, Jean-Louis Pujol

**Affiliations:** 1 Department of Biochemistry, Nantes University Hospital, 9 quai Moncousu, 44093 Nantes, France; guillaume.herbreteau@chu-nantes.fr; 2 IFCT Intergroupe Francophone de Cancérologie Thoracique, 10 Rue de la Grange Batelière, 75009 Paris, France; alexandra.langlais@ifct.fr (A.L.); franck.morin@ifct.fr (F.M.); 3 Department of Multidisciplinary Oncology and Therapeutic Innovations, Assistance Publique—Hôpitaux de Marseille, Aix Marseille University, 13015 Marseille, France; laurent.GREILLIER@ap-hm.fr; 4 Department of Thoracic Oncology, 54 Rue Henri Sainte Claire Deville, CHITS CH Sainte Musse, 83000 Toulon, France; clarisse.audigier-valette@ch-toulon.fr; 5 Institut de Cancérologie de Lorraine Alexis Vautrin, 6 Avenue de Bourgogne, 54519 Vandoeuvre-les-Nancy, France; l.uwer@nancy.unicancer.fr; 6 Pôle Hippocrate, Angers University Hospital, 49933 Angers, France; jose.hureaux@chu-angers.fr; 7 Thoracic Oncology Unit, CHU Grenoble Alpes, 38700 Grenoble, France; DMoro-Sibilot@chu-grenoble.fr; 8 Department of Pneumology, Thoracic Oncology and Respiratory Intensive Care, Rouen University Hospital, 76000 Rouen, France; florian.guisier@chu-rouen.fr; 9 Service de Pneumologie CHRU Hôpitaux de Tours, Hôpital Bretonneau, 2 Boulevard Tonnellé, 37000 Tours, France; D.CARMIER@chu-tours.fr; 10 Service de Pneumologie, CHU Caen Normandie, Av de La Côte de Nacre, 14000 Caen, France; madelaine-j@chu-caen.fr; 11 Pôle Médecine, Centre Antoine Lacassagne, 33 Avenue de Valombrose, 06100 Nice, France; josiane.otto@nice.unicancer.fr; 12 Service de Pneumologie Aiguë Spécialisée et Cancérologie Thoracique, Centre Hospitalier Lyon Sud, 165 Chemin du Grand Revoyet, 69310 Pierre-Benite, France; pierre-jean.souquet@chu-lyon.fr; 13 Department of Thoracic Oncology, Bichat Claude Bernard Hospital, 75018 Paris, France; valerie.gounant@aphp.fr; 14 Service de Pneumologie, 58 Rue Montalembert, CHU G Montpied, 63000 Clermont Ferrand, France; pmerle@chu-clermontferrand.fr; 15 Service de Pneumologie, Centre Hospitalier, 194 Avenue Rubillard, 72037 Le Mans, France; omolinier@ch-lemans.fr; 16 Service de Pneumologie, Centre Hospitalier, 4 Boulevard Hauterive, 64000 Pau, France; aldo.renault@ch-pau.fr; 17 Service de Pneumologie, Centre Hospitalier, Université Paul Sabatier, 31300 Toulouse, France; rabeau.a@chu-toulouse.fr; 18 Department of Biochemistry, Nantes University Hospital, 9 Quai Moncousu, 44093 Nantes, France; marc.denis@chu-nantes.fr; 19 Department of Thoracic Oncology, Montpellier Regional University Hospital, 34090 Montpellier, France

**Keywords:** SCLC, ctDNA, atezolizumab, mutation, *TP53*, *RB1*, *NOTCH*

## Abstract

Background: The IFCT-1603 trial evaluated atezolizumab in small cell lung cancer (SCLC). The purpose of the present study was to determine whether circulating tumor DNA (ctDNA), prospectively collected at treatment initiation, was associated with the prognosis of SCLC, and whether it identified patients who benefited from atezolizumab. Methods: 68 patients were included in this study: 46 patients were treated with atezolizumab and 22 with conventional chemotherapy. Circulating DNA was extracted from plasma and NGS (Next Generation Sequencing) looked for mutations in the *TP53, RB1*, *NOTCH1, NOTCH2,* and *NOTCH3* genes. ctDNA was detectable when at least one somatic mutation was identified, and its relative abundance was quantified by the variant allele fraction (VAF) of the most represented mutation. Results: We found that 49/68 patients (70.6%) had detectable baseline ctDNA. The most frequently identified mutations were TP53 (32/49; 65.3%) and RB1 (25/49; 51.0%). Patients with detectable ctDNA had a significantly lower disease control rate at week 6 compared with patients with no detectable ctDNA, regardless of the nature of the treatment. Detection of ctDNA was associated with a poor OS prognosis. The detection of ctDNA at a relative abundance greater than the median value was significantly associated with poor overall survival (OS) and progression free survival (PFS). Interestingly, the benefit in overall survival (OS) associated with low ctDNA was more pronounced in patients treated with atezolizumab than in patients receiving chemotherapy. Among patients whose relative ctDNA abundance was below the median, those treated with atezolizumab tended to have higher OS than those in the chemotherapy arm. Conclusion: ctDNA is strongly associated with the prognosis of SCLC patients treated with second-line immunotherapy. Its analysis seems justified for future SCLC clinical trials.

## 1. Introduction

Small cell lung cancer accounts for 14% of all lung cancers. It remains a major challenge for oncology as the advances made over the past three decades have been modest [1]. Despite the recent introduction of immunotherapy, chemotherapy remains the backbone of SCLC therapy [2]. Notwithstanding the high chemosensitivity of SCLC, survival is low due to secondary chemoresistant relapses. In this second-line setting, the survival of patients treated either with reinduction chemotherapy or single-drug topotecan or amrubicin is approximately 33 weeks [3].

Combining chemotherapy with immune checkpoint inhibitors (ICPI) is rapidly becoming the standard first-line treatment for extensive-disease small cell lung cancer (ED-SCLC). Three randomized phase 3 trials have shown that adding monoclonal antibodies (Ab) against programmed death 1 (PD1) and its ligand (PD-L1) to conventional platinum-etoposide chemotherapy prolonged both progression-free survival (PFS) and overall survival (OS) for ED-SCLC patients [4,5,6]. The hazard ratio (HR) of the risk of death for patients receiving combined immunotherapy and chemotherapy was around 0.60. The overall survival benefit was significant for atezolizumab [4] and durvalumab [5], but not for pembrolizumab [6]. In contrast with the demonstrated outcome improvement induced by combining ICPI with chemotherapy in the first-line setting, immunotherapy failed to improve survival of SCLC patients in the second-line setting. Several anti-PD1 and anti-PD-L1 Abs have been compared to second-line chemotherapy for patients who relapsed after first-line chemotherapy in various randomized trials: Neither atezolizumab [7], nor nivolumab [8] improved survival in the second-line setting. Moreover, nivolumab combined with ipilimumab (an Ab against cytotoxic T-lymphocyte-associated protein 4 (CTLA4)) failed to achieve a maintenance goal in a large randomized trial including patients with ED-SCLC [9]. Recently, in limited-disease SCLC patients who had received concomitant radio-chemotherapy followed by prophylactic cranial irradiation, the initial results of the STIMULI trial showed that consolidation with combined nivolumab and ipilimumab failed to improve PFS compared to observation alone [10].

The efficiency of combined immunotherapy and chemotherapy in the first line might be regarded as a synergistic effect of the two therapies (chemotherapy inducing damage-associated molecular patterns that enhance CD8 response to ICPI), but also as a reduction of the risk of hyper-progression, and as the better performance index and immunity status of patients who are still treatment-naïve. The same can be said for the second-line setting. In this setting, the very short PFS (usually less than 2 months) is a deterrent to proposing an ICPI to a patient with relapse, but in each trial, one can observe a significant subgroup of patients (around 10%) with long-term survival (over 2 years), an observation extremely rare with second-line topotecan or second-line amrubicin. Neither PD-L1 tissue expression nor mutational tumor burden were able to discriminate a subgroup of patients with a maximized benefit in first and second line setting during the various aforementioned randomized trials.

In 2019, we published the IFCT-1603 trial, an open-label randomized non-comparative Phase 2 study that sought to evaluate atezolizumab activity as systemic therapy in SCLC progressing after first-line platinum/etoposide-based chemotherapy [7]. In this study, 1200 mg atezolizumab monotherapy given once every three weeks resulted in a 2.3% response rate and a 1.4-month median PFS, thus precluding initiation of the Phase 3 study part. Again, neither PD-L1 tissue expression nor any other marker was associated with survival in this trial. In order to conduct an ancillary study, plasma samples were prospectively collected at the initiation of treatment (atezolizumab or conventional chemotherapy) to analyze circulating tumor DNA.

Circulating tumor DNA (ctDNA) corresponds to the fraction of cell-free plasma DNA of tumor origin: ctDNA can be detected and quantified by identifying in circulating DNA the same somatic mutations as those found in tumor tissue [11]. It is now well-established that the amount of ctDNA is correlated with tumor stage and burden, and the detection of ctDNA constitutes an independent prognostic factor in many cancers (non-small cell lung cancer (NSCLC) [12], melanoma [13], colorectal cancer [14], etc.). In SCLC, ctDNA can be easily detected by analyzing a limited number of genes, since almost all SCLCs carry mutations in TP53 and RB1 genes, and the genes of the NOTCH family show a mutation in approximately 25% of cases [15]. A few studies have shown that high pre-treatment ctDNA levels were associated with a poor prognosis in PFS and OS in SCLC, but no study has evaluated its relevance for treatment decision [16,17,18].

The present ancillary study of the IFCT-1603 trial was aimed at determining whether (i) ctDNA was associated with the prognosis of SCLC in the IFCT-1603 trial, and (ii) whether this biomarker made it possible to distinguish groups of patients for whom atezolizumab showed better efficacy.

## 2. Patients and Methods

### 2.1. Summary of the IFCT-1603 Trial

In this study, patients were randomized 2:1 to atezolizumab, 1200 mg IV, every three weeks until progression or unacceptable toxicity, or conventional CT up to six cycles: In this chemotherapy group, patients received oral or IV topotecan or re-induction of carboplatin-etoposide doublet according to the investigator’s choice. The main eligibility criteria were performance status (PS) 0–2 and measurable disease (RECIST -Response Evaluation Criteria In Solid Tumours- 1.1). We performed no selection on PD-L1 tissue expression. Patients receiving corticosteroid therapy, patients with a history of autoimmune disease, and those with active brain metastases were excluded. The primary endpoint was an objective response rate at the sixth week in eligible patients with confirmation needed at 12 weeks. Patients were stratified (minimization) by PS (0–1 versus 2), limited versus extensive disease at the time of randomization, gender, performance status, and presumed disease sensitivity (refractory versus sensitive disease, the latter defined as relapse more than 90 days after ending first-line therapy). PD-L1 expression was assessed centrally in archival or fresh tumor samples according to previously published scoring criteria. This non-comparative study used a two-stage design with a 2:1 randomization and O’Brien–Fleming stopping rules, allowing for early stopping for futility. A response rate lower than 15% was considered as unacceptable and a response rate equal to or higher than 33% was considered as a good response rate. The null hypothesis was rejected if 12/45 patients or more were responders.

All subjects gave their informed consent for inclusion before they participated in the study. The study was approved by an institutional review board (Sud – Mediterranée IV, Montpellier University, France) and was registered on clinicaltrials.gov under trial number NCT03059667.

From March 2017 to December 2017, 73 patients were accrued by 24 French institutions. Forty-nine patients were randomly allocated to the atezolizumab group and 24, to the chemotherapy group; six patients in the atezolizumab group were ineligible due to brain metastases (*n* = 3) or corticosteroid doses higher than 10 mg equivalent prednisolone during the previous month (*n* = 3). One patient in the atezolizumab group did not receive any treatment due to rapid impairment of clinical status. Forty-five out of the 49 atezolizumab patients were withdrawn for death or progressive disease, and three patients continued therapy versus none in the chemotherapy group. Demographic and baseline characteristics were well-balanced between the two groups: In the atezolizumab group, 83% of patients had a PS 0–1, 80% had an extensive disease, and 67% had a sensitive relapse.

In the atezolizumab group, one of the 43 eligible patients achieved an objective response at the sixth week, giving a 2.3% objective response (95% confidence interval (CI): 0–6.8%). Eight other patients had a stable disease resulting in a disease control rate of 21% (95% CI: 8.8–33%). Median follow-up was 13.7 months (95% CI: 12.7-not reported). Median PFS in the intention-to-treat population significantly differed between the two groups (1.4 months (95% CI: 1.2–1.5) versus 4.3 months (95% CI: 1.5–5.9) in the atezolizumab and chemotherapy groups, respectively; adjusted hazard ratio (HR) atezolizumab arm = 2.26 (95% CI: 1.30–3.93); *p* = 0.004). The 6-month PFS for atezolizumab was 6.3% (95% CI: 0.0–13.1%). Random allocation had no effect upon OS since the date of randomization: Median OS: 9.5 months (95% CI: 3.2–14.4) versus 8.7 months (95% CI: 4.1–12.7) for the atezolizumab and chemotherapy group, respectively; adjusted HR atezolizumab arm = 0.84 (95% CI: 0.45–1.58); *p* = 0.60. The 1-year OS rate for the atezolizumab group was 42.5% (95% CI: 26.9–58.2%). Exploratory subgroup analyses of unstratified HRs were conducted according to gender, performance status (0–1 versus 2), disease sensitivity (refractory versus sensitive), and disease stage (limited versus extensive). We did not identify features of a favorable effect with atezolizumab. PD-L1 expression was evaluable in 54 patients: PD-L1 was expressed by less than 1% of the tumor cells in 53/54 patients (98.2%), and only one patient (1.8%) showed PD-L1 tumor expression between 5% and 50%. This low PD-L1 expression precluded any subgroup analyses.

### 2.2. ctDNA Analysis

Before initial treatment administration, plasma samples were collected in EDTA tubes, centrifuged at 2000× *g* for 10 min, decanted and frozen at −80 °C within 4 h of collection. Circulating DNA was extracted from EDTA plasma using the Maxwell^®^ RSC Large-Volume ccfDNA Plasma Kit (Promega), and eluted in 60 μL of elution buffer as recommended by the supplier. The circulating DNA concentration was quantified for each sample using a Quantus Fluorometer (Promega, Charbonnières-les-Bains, France).

NGS libraries were made using a QIAseq Targeted DNA Custom Panel (QIAGEN, Courtaboeuf, France) Kit, an amplicon library construction kit based on Anchored Multiplex PCR (AMP) technology (https://www.qiagen.com/us/products/discovery-and-translational-research/next-generation-sequencing/dna-sequencing/somatic-panels/qiaseq-targeted-dna-custom-panels). This panel targeted all exons of *TP53*, *RB1*, *NOTCH1*, *NOTCH2*, and *NOTCH3* genes (genomic positions of AMP primers available on demand). NGS libraries were created in accordance with the supplier’s guidelines, then sequenced on a MiSeq sequencer (Illumina, Paris, France).

NGS data were interpreted in CLC Genomics Workbench 12.0 (QIAGEN). Unmapped reads, in FASTQ format, were trimmed for adapter sequences. Reads were then mapped to human genome 38 (hg38) and realigned taking into account the identified insertions and deletions. Mapped reads were then trimmed for AMP primer sequences. Finally, variants were called using the base-position error rate (BPER) analysis as described by Pécuchet et al. [19]. Briefly, this method statistically compares the allelic frequency of each variant identified among the mapped reads with the sequencing error rate, determined beforehand for each genomic position on negative control circulating DNA samples taken from a cohort of 28 healthy donors.

Patients were split into two subgroups, depending on whether or not one (or more) circulating DNA mutation(s) were identified. Mutations found in circulating DNA were described for patients with at least one detectable mutation, and the relative abundance of ctDNA in plasma was estimated by the variant allele fraction (VAF) of these mutations (in cases where several mutations were identified, the highest VAF was considered).

### 2.3. Statistics

For each factor, a descriptive analysis and survival analysis (including a prognostic analysis) were performed. The between-group distribution of qualitative variables was compared using the χ^2^ test or Fisher’s exact test.

Overall survival was defined as the time from randomization to the date of death, and PFS was defined as the time from randomization to either RECIST 1.1 disease progression or death from any cause, whichever occurred first. Patients who did not progress when the first subsequent treatment was started were censored on that date. Patients who did not progress and who had not received subsequent treatment at the cutoff date were censored on the date of their last assessment.

Probability of survival was estimated using the Kaplan–Meier method, with survival differences analyzed using log-rank tests. Hazard ratios (HRs) and 95% confidence intervals (CIs) were estimated using a Cox model. The HR for risk of death, along with the HR for risk of progression were calculated (i) without adjustment, (ii) adjusted for stratification factors (i.e., limited versus extensive disease at the time of randomization, gender, performance status and, sensitive versus refractory disease), and (iii) adjusted for stratification factors and unbalanced factors between groups (variables to be tested in the model were selected based on univariate analysis results (*p* < 0.20)). A *p*-value of less than 0.05 was considered statistically significant. SAS software version 9.3 (Brie Comte Robert, France) was used.

## 3. Results

### 3.1. Patients

From March to December 2017, 73 patients were included by 24 French institutions. Among these, 68 patients were included in the present study. Five patients were excluded because no plasma was collected before the first administration of treatment. Forty-six patients were included in the atezolizumab arm and 22 in the chemotherapy arm. Demographic characteristics were well-balanced between both groups (Supplementary Table S1). Overall, the median age was 64.5 years, 86.7% had a performance status of 0 to 1, 76.5% had extensive disease, and 64.7% had sensitive relapse (progression at least 90 days after the last dose of first-line chemotherapy) (Table 1). The data cutoff took place on 30 June 2018.

### 3.2. ctDNA Analysis

Overall, mutations in *TP53*, *RB1*, and *NOTCH1*–*3* genes were identified in baseline circulating DNA in 49 out of 68 patients (70.6%). The most frequently identified mutations were *TP53* (32/49; 65.3%) and *RB1* (25/49; 51.0%) loss-of-function mutations. In total, mutations of *TP53, RB1*, or both were found in the vast majority of patients for whom at least one mutation was detectable in circulating DNA (44/49; 89.8%). Almost a quarter of patients had at least one mutation in one of the *NOTCH* genes (12/49; 24.5%).

Patient characteristics were comparable between patients with a mutation detected in circulating DNA and those without a detectable mutation (Table 1).

### 3.3. Response and Survival

Patients with a detectable circulating mutation had a significantly lower disease control rate at week 6 (objective response or stable disease) than patients with no detectable mutation, regardless of the nature of the treatment (29.5% versus 58.8%, respectively; *p* = 0.030). By treatment subgroup, the detection of a mutation in circulating DNA was not associated with any difference in disease control rate in patients treated with chemotherapy (64.3% versus 71.4%; *p* = 0.672), while patients treated with immunotherapy had a significantly lower disease control rate when a circulating mutation was detected (13.3% versus 50%; *p* = 0.0145).

**Figure 1:**
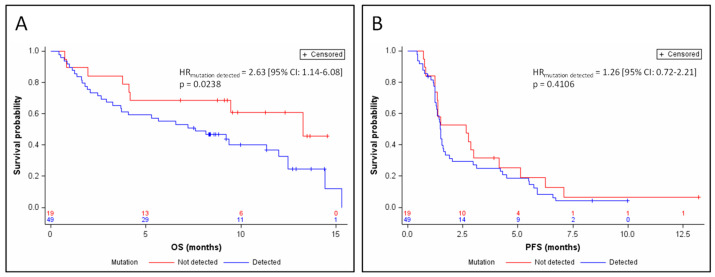
Kaplan–Meier estimates of overall survival (OS) (**A**) and progression-free survival (PSF) (**B**), depending on the detection of ctDNA. HR, hazard ratio.

Median follow-up was 11.4 months. Detection of a mutation in circulating DNA was associated with a poor prognosis in OS (HR_mutation detected_ = 2.63 (95% CI: 1.14–6.08) *p* = 0.0238; Figure 1A) after adjustment for the stratification factors (sensitive relapse, performance status, stage, and gender). Median OS was 7.6 months in the presence of a detectable mutation, versus 13.3 months in the absence of a detectable circulating mutation. This prognostic value was independent of the nature of the treatment, after adjustment for the stratification factors and treatment arm (HR_mutation detected_ = 2.60 (95% CI: 1.11–6.08) *p* = 0.0272). The detection of a mutation in circulating DNA was not correlated with PFS (HR_mutation detected_ = 1.26 (95% CI: 0.72–2.21) *p* = 0.4106; Figure 1B). None of the mutated genes taken individually showed any association with PFS or OS.

**Figure 2:**
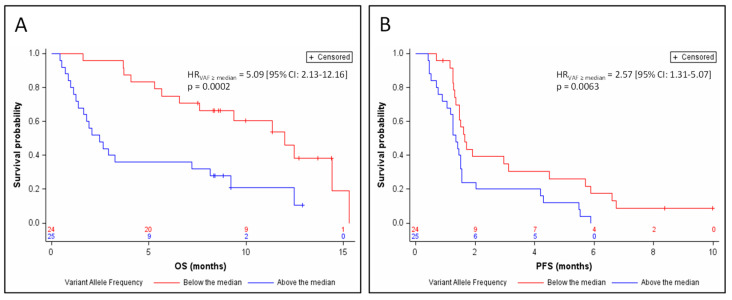
Kaplan–Meier estimates of overall survival (**A**) and progression-free survival (**B**), depending on relative ctDNA abundance (below or above the median).

The relative abundance of tumor DNA in plasma was estimated by the variant allele fraction (VAF) of the somatic mutations identified in circulating DNA (in cases where several mutations were identified, the highest VAF was considered). Among the samples from 49 patients for whom a somatic mutation of *TP53*, *RB1*, or *NOTCH1*–*3* was identified in circulating DNA, ctDNA represented 2.8% to 95.5% of the total amount of circulating DNA, with a median relative abundance = 44.5%. The detection of ctDNA at a relative abundance greater than this median value was significantly associated with a poor prognosis in terms of OS (HR_VAF≥median_ = 5.09 (95% CI: 2.13–12.16) *p* = 0.0002; Figure 2A) and of PFS (HR_VAF≥median_ = 2.57 (95% CI: 1.31–5.07) *p* = 0.0063; Figure 2B) after adjustment for the stratification factors. Median OS was 2.5 months for patients whose ctDNA was at relative abundance greater than the median (95% CI: 1.4–8.1), versus 12 months for patients whose ctDNA was detectable at less than or equal to the median relative abundance (95% CI: 6.6–15.3). Median PFS was 1.3 months for patients whose ctDNA was above the median relative abundance (95% CI: 1.1–1.5), versus 1.6 months for patients whose ctDNA was detectable at relative abundance less than or equal to the median (95% CI: 1.3–3.1). The prognostic value of relative ctDNA abundance was independent of the nature of the treatment, after adjustment for the stratification factors and treatment arm (OS: Adjusted HR_VAF≥median_ = 5.59 (95% CI: 2.30–13.58) *p* = 0.0001; PFS: Adjusted HR_VAF≥median_ = 2.94 (95% CI: 1.48–5.83) *p* = 0.0020). Interpreted as a continuous variable, relative ctDNA abundance remained a prognostic factor in OS and PFS according to a Cox model adjusted on the stratification factors (for OS: HR = 1.033 (95% CI: 1.016–1.05) *p* = 0.0001; for PFS: HR = 1.015 (95% CI: 1.003–1.027) *p* = 0.0163).

### 3.4. Efficacy of Atezolizumab, Based on ctDNA Data

**Figure 3:**
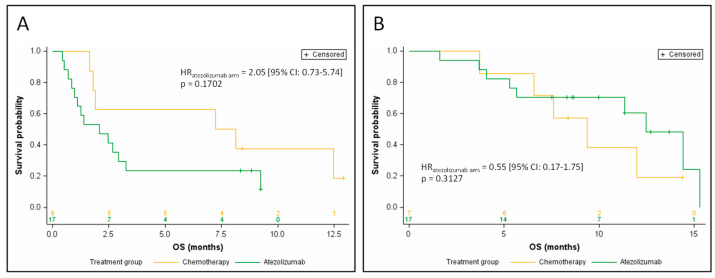
Kaplan–Meier estimates of overall survival, depending on the treatment arm, for patients with relative ctDNA abundance above the median (**A**), and for patients with relative ctDNA abundance below the median (**B**).

In the 25 patients with relative ctDNA abundance greater than the median, 8 patients were treated with chemotherapy, and 17 patients were treated with atezolizumab. In this subgroup, there was a trend toward a shorter OS in atezolizumab-treated patients when compared with chemotherapy-treated patients in univariate analysis (HR_atezolizumab arm_ = 2.05 (95% CI: 0.73–5.74) *p* = 0.1702; Figure 3A). The median OS was 2.1 months for the atezolizumab arm versus 7.7 months for the chemotherapy arm.

Conversely, in the 24 patients whose relative ctDNA abundance was below or equal to the median (7 patients treated with chemotherapy, 17 patients treated with atezolizumab), patients treated with atezolizumab had a longer OS than in the chemotherapy arm in univariate analysis, although the difference did not reach statistical significance (HR_atezolizumab arm_ = 0.55 (95% CI: 0.17–1.75) *p* = 0.3127; Figure 3B). Median OS was 9.0 months for the atezolizumab arm versus 5.0 months for the chemotherapy arm.

**Figure 4:**
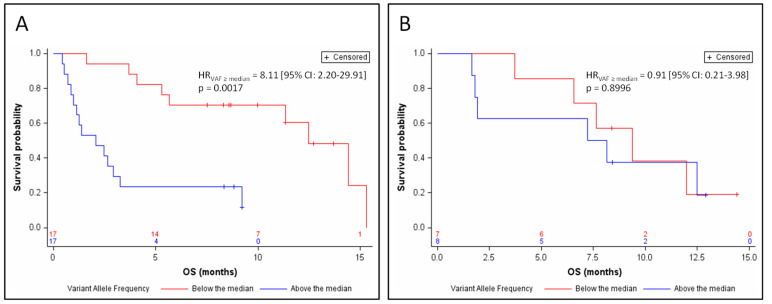
Kaplan–Meier estimates of overall survival, depending on relative ctDNA abundance (above or below the median) for patients treated with atezolizumab (**A**) or chemotherapy (**B**).

By treatment subgroup, the benefit in OS associated with the low ctDNA abundance was clearly more pronounced in patients treated with atezolizumab (median OS = 12.5 months versus 2.1 months for patients with low and high ctDNA abundance, respectively; HR_VAF≥median_ = 8.11 (95% CI: 2.20–29.91) *p* = 0.0017; Figure 4A) than in patients receiving chemotherapy (median OS = 9.4 months versus 7.7 months for patients with low and high ctDNA abundance respectively; HR_VAF≥median_ = 0.91 (95% CI: 0.21–3.98) *p* = 0.8996; Figure 4B) after adjustment for stratification factors.

## 4. Discussion

Our study demonstrated that the detection of ctDNA, especially in high abundance, was associated with reduced therapeutic response and poor prognosis with shorter PFS and OS, independently of SCLC main prognostic factors (stage, performance status, resistant disease), and especially in patients treated with an anti-PD-L1 immunotherapy. The size of the chemotherapy arm was insufficient (*n* = 22) to demonstrate any association between ctDNA and disease control rate or prognosis in chemotherapy-treated patients.

A few studies have evaluated the prognostic value of ctDNA in SCLC. Almodovar et al. studied circulating DNA from 27 SCLC patients and showed genomic landscapes similar to those observed in our study [16]. Twenty out of 27 patients (74.1%) had at least one detectable *TP53*, *RB1*, or *NOTCH1–3* mutation in circulating DNA. The most frequent mutations were TP53 mutations (70.4%) and *RB1* mutations (51.9%). In this study, a quantity of ctDNA (defined in genomic equivalents) greater than the median value was associated with poor OS after adjustment for stage and treatment (HR = 2.73 (95% CI: 1.27–5.86) *p* = 0.0099). In another study on the genomic landscape and subclonal architecture of SCLC based on ctDNA analysis, Nong et al. also showed mutations similar to those we identified in our study [17]. At least one mutation of TP53, RB1, or was detectable for 21 out of 22 patients (95.5%), including a majority of *TP53* mutations (90.9%) and *RB1* mutations (63.6%). Up to 90.9% of patients presented a mutation of *TP53, RB1*, or both, and *NOTCH1–3* mutations were found in 27.3% of patients. Furthermore, Nong et al. showed that the amount of ctDNA was significantly associated with PFS and OS. In this study, among patients with higher versus lower than median ctDNA abundance, median PFS was 5.3 months (95% CI: 5.0–5.6) versus 10.0 months (95% CI: 9.5–10.5 months), and median OS was 9.3 months (95% CI: 1.6–17.0) versus 25.0 months (95% CI: 4.8–45.2 months). These findings are consistent with our results, although Nong et al. quantified relative ctDNA abundance by the mean VAF of identified mutations rather than the highest VAF, giving lower ctDNA levels and cut-off than those we observed (median relative ctDNA abundance = 18% versus 44.5% in our study). In a recent study by Mohan et al., at least one mutation of our panel was identified in 51 out of 62 patients (82.3%) [18]. Mutations in *TP53*, *RB1*, and *NOTCH1–3* were identified in 79.0%, 32.3%, and 21.0% of patients, respectively. Relative ctDNA abundance was assessed by the highest VAF similarly to our study. A high ctDNA level, considering a cut-off of 37%, was associated with poor OS (HR = 2.66 (95% CI: 1.39–5.11) *p* = 0.0003).

The association between survival and ctDNA amount could be explained by the fact that ctDNA is quantitatively correlated with tumor burden and tumor aggressiveness. ctDNA constitutes a highly specific tumor biomarker for many cancers, quantitatively associated with tumor burden as assessed by CT scan [20], and with tumor metabolism measured by PET-scan [21]. In SCLC, relative ctDNA abundance was shown to be moderately correlated with tumor burden as assessed by imaging [17]. It has been suggested that ctDNA may better quantify the tumor burden than imaging, as CT scan may be limited for the detection and measurement of small metastases, particularly common in SCLC. The study of another circulating tumor biomarker, circulating tumor cells (CTCs), would appear to validate this hypothesis: SCLC is the cancer with the higher level of CTCs [22], and despite a lower detection sensitivity than that observed for ctDNA [16,18], several studies have shown that this biomarker behaves similarly to ctDNA: The number of CTCs is quantitatively correlated with tumor mass, and a high number of CTCs at the baseline is associated with poor PFS and OS [18,23,24,25,26,27,28,29].

The study herein was based on the phase II trial IFCT-1603, which evaluated the activity of atezolizumab, an anti-PD-L1 antibody, as a systemic therapy in SCLC progressing after first-line platinum-etoposide-based chemotherapy [7]. Although the IFCT-1603 trial failed to demonstrate efficacy of atezolizumab in this indication, we observed that the baseline ctDNA level allowed the definition of subgroups for which the efficacy of atezolizumab appeared to differ radically: In patients with high baseline ctDNA levels, atezolizumab appeared to be less effective than chemotherapy, while atezolizumab tended to be more effective than chemotherapy in patients with low ctDNA concentration at baseline. Despite the fact that the size of these two subgroups was insufficient in our study to be able to demonstrate a significant benefit of atezolizumab in a subgroup, it is remarkable and unprecedented in SCLC that a biomarker was able to distinguish such a difference in efficacy of an immunotherapy between two subgroups. In contrast, in the IFCT-1603 trial, 98.2% of patients had less than 1% tumor cells expressing PD-L1, and no patient characteristics were associated with atezolizumab efficacy. In addition, neither the tissue expression of PD-L1 nor the mutational tumor load was able to discriminate a subgroup associated with improved efficacy of first- and second-line immunotherapies in any clinical trial in SCLC [4,5,6,7,8,9].

Our study deserves further evaluation of the prognostic and predictive value of ctDNA in therapeutic trials, and in particular those testing the efficacy of immunotherapy of SCLC, in order to confirm our findings and identify subgroups of patients able to draw maximum benefit from these treatments.

## Figures and Tables

**Table 1:** Patient characteristics based on the detectability of ctDNA.

			Total (*n* = 68)	Mutation Detected (*n* = 49)	No Mutation Detected (*n* = 19)	*p*
Age (years)		Mean ± SD	65.14 +/− 7.28	65.34 +/− 7.56	64.62 +/− 6.68	0.6718
		Median	64.54	65.44	63.03	
		Range	(51.11–85.47)	(51.11–85.47)	(57.2–85.05)	
Gender	Female	*n* (%)	29 (42.6)	19 (38.8)	10 (52.6)	0.2999
	Male	*n* (%)	39 (57.4)	30 (61.2)	9 (47.4)	
Performance status	0	*n* (%)	26 (38.2)	18 (36.7)	8 (42.1)	0.3656
	1	*n* (%)	33 (48.5)	26 (53.1)	7 (36.8)	
	2	*n* (%)	9 (13.2)	5 (10.2)	4 (21.1)	
Smoker (current or former)	Yes	*n* (%)	66 (97.1)	48 (98)	18 (94.7)	0.4838
	No	*n* (%)	2 (2.9)	1 (2)	1 (5.3)	
Stage at time of random allocation	Limited disease	*n* (%)	16 (23.5)	10 (20.4)	6 (31.6)	0.3530
	Extensive disease	*n* (%)	52 (76.5)	39 (79.6)	13 (68.4)	
Sensitive relapse (progression ≥90 days after last first-line chemotherapy dose)	Yes	*n* (%)	44 (64.7)	32 (65.3)	12 (63.2)	0.8679
	No	*n* (%)	24 (35.3)	17 (34.7)	7 (36.8)	
Mutated gene	*TP53*	*n* (%)	-	32 (65.3)	-	-
	*RB1*	*n* (%)	-	25 (51)	-	
	*NOTCH1*	*n* (%)	-	3 (6.1)	-	
	*NOTCH2*	*n* (%)	-	6 (12.2)	-	
	*NOTCH3*	*n* (%)	-	4 (8.2)	-	
Treatment arm	Chemotherapy	*n* (%)	22 (32.4)	15 (30.6)	7 (36.8)	0.6222
	Atezolizumab	*n* (%)	46 (67.6)	34 (69.4)	12 (63.2)	

## Supplementary Materials

The following are available online at https://www.mdpi.com/2077-0383/9/12/3861/s1, Table S1: Patient characteristics by treatment arm.
