# Supplementary material for: Circulating Tumor DNA as a Prognostic Determinant in Small Cell Lung Cancer Patients Receiving Atezolizumab
Source: J Clin Med. 2020 Nov 27;9(12):3861. doi: 10.3390/jcm9123861 (PMC7760916; doi:10.3390/jcm9123861)
Supplement: Supplementary file 1 [file jcm-09-03861-s001.pdf]

Supplementary table S1. Patient characteristics by treatment arm.

			**Total (N = 68)**	**Chemotherapy (N = 22)**	**Atezolizumab (N = 19)**	***p***
Age (years)		Mean ± SD	65.14 +/− 7.28	62.79 +/− 6.00	65.14 +/− 7.63	0.08
		Median	64.54	62.99	65.85	
		Range	(51.1-85.5)	(51.8-79.1)	(51.1-85.5)	
Gender	Female	N (%)	29 (42.6)	10 (45.5)	19 (41.3)	0.75
	Male	N (%)	39 (57.4)	12 (54.4)	27 (58.7)	
Performance Status	0	N (%)	26 (38.2)	7 (31.8)	19 (41.3)	0.51
	1	N (%)	33 (48.5)	13 (59.1)	20 (43.5)	
	2	N (%)	9 (13.2)	2 (9.1)	7 (15.2)	
Smoker (current or former)	Yes	N (%)	66 (97.1)	22 (100)	44 (95.7)	1.00
	No	N (%)	2 (2.9)	0	2 (2.9)	
Stage at time of random allocation	Limited disease	N (%)	16 (23.5)	7 (31.8)	9 (19.6)	0.26
	Extensive disease	N (%)	52 (76.5)	15 (68.2)	37 (80.4)	
Sensitive relapse (progression ≥ 90 days after last first-line chemotherapy dose)	Yes	N (%)	44 (64.7)	14 (63.6)	30 (65.2)	0.90
	No	N (%)	24 (35.3)	8 (36.4)	16 (34.8)	
